# Supplementary material for: Atomistic Modeling of Functionalized Magnetite Surfaces with Oxidation States
Source: J Phys Chem Lett. 2025 Jun 24;16(26):6765–70. doi: 10.1021/acs.jpclett.5c00679 (PMC12235627; doi:10.1021/acs.jpclett.5c00679)
Supplement: Supplementary file 1 [file jz5c00679_si_001.pdf]

Supporting Information: Atomistic modeling of functionalized magnetite surfaces with oxidation states

Emre Gürsoy,[†] Robert H. Meißner,[†,‡] and Gregor B. Vonbun-Feldbauer[*,†,¶]

†*Institute for Interface Physics and Engineering, Hamburg University of Technology, 21073 Hamburg, Germany*

‡*Institute of Surface Science, Helmholtz-Zentrum Hereon, 21502 Geesthacht, Germany*

¶ *Institute of Advanced Ceramics, Hamburg University of Technology, 21073 Hamburg, Germany*

E-mail: gregor.feldbauer@tuhh.de

Contents

Simulation settings 2

Oxidation state minimization 2

Magnetite and carboxylic acid force fields 3

Electron Localization Function 5

Simulation settings

All force-field simulations were performed using LAMMPS.[1] Equations of motions were numerically integrated with a timestep of 0.5 fs in the MD simulations using the commonly employed velocity-Verlet algorithm. Long-range Coulomb interactions were treated with a PPPM[2] solver with an (relative) accuracy of 10^{-6} and a real-space cutoff of 12 Å. Since we are dealing with an infinitely extended slab-like system to represent a real magnetite surface, long-range Coulombic interactions have been calculated using the dipole correction method[3] to avoid slab-slab interactions over the non-periodic dimension. Pairwise interaction between bonded atom pairs were scaled down following the common AMBER scaling rules.[4]

The Density Functional Theory (DFT) calculations follow the setup of our previous studies.[5,6] The Vienna Ab Initio Simulation Package (VASP, version 5.4.4)[7–10] with the PBE+U approach[11,12] ($U_{\text{eff}} = 4\,\text{eV}$ on Fe d-electrons) and PAW pseudopotentials[13] was employed for investigating adsorption at magnetite surfaces. The formal oxidation states of the ions are approximated based on Bader charges[6,14] obtained with the tools from the Henkelman group.[15–17] As pointed out in our previous publication,[6] the system size and symmetry used in the calculations can significantly effect the charges, their distributions and thus the oxidation states. Therefore, slabs up to 25 layers were used here and symmetries other than time-reversal symmetry were disabled in the calculations. However, because of computational limitations and convergence issues for larger systems, no results for thicker slabs are presented.

Oxidation state minimization

The cyclic process of oxidation state minimization starts by applying energy minimization on the adsorbates, modifying their geometry, followed by oxidation state swaps, forcing Fe ions to adapt to the modified adsorbate geometry. In the first energy minimization-oxidation state swap cycle, we start with a high $T^{\mathrm{MC}} = T_0^{\mathrm{MC}}$ to explore the configuration space freely. We then gradually lower T^{MC} using an exponential decrease function, $T_\lambda^{\mathrm{MC}} = T_0^{\mathrm{MC}} \cdot \eta^\lambda$, with $\eta = 0.9$. This approach is commonly known as simulated annealing[18] and often used for sampling configuration spaces effectively. For the initial annealing temperature we chose $T_0^{\mathrm{MC}} = 10^5\,\mathrm{K}$, which roughly translates to a swap probability of 0.9 for magnetite slabs we used (up to 65 layers). For thicker slabs, however, T_0^{MC} should be adjusted to yield approximately 0.9 swap probability. At each cycle, n_{swaps} swap attempts are made. Assuming that at room temperature all Fe_{tet} ions on the (001) surfaces have Fe^{3+} oxidation state,[6] we restricted the swaps to Fe_{oct} ions only. Moreover, in our initial models where we did not restrict swaps to Fe_{oct} ions, we observed the same oxidation state minimized structure as in the restricted case. However, in (111) or assumable in other magnetite surfaces or at elevated temperatures where Fe^{2+}_{tet} ions are present,[6] all Fe ions should be swapped. The number of swaps was carefully chosen so that each Fe^{2+}_{oct} ion has a chance to be swapped with another Fe^{3+}_{oct} ion at any given T^{MC}. In stoichiometric magnetite, half of the Fe_{oct} ions are in Fe^{2+} and the other half are in Fe^{3+} oxidation state, thus there are $1/4\,(n_{\mathrm{Fe_{oct}}})^2$ possible oxidation state swaps. So we chose $n_{\mathrm{swaps}} = 1/4\,(n_{\mathrm{Fe_{oct}}})^2$. Because of the annealing scheme used here, there were in total 108 cycles of energy minimization-oxidation state swaps.

Magnetite and carboxylic acid force fields

Force field parameters used for magnetite and surface hydroxyls are taken from ClayFF,[19] with the exception of Fe^{2+}, which is not included in ClayFF, thus taken from Gürsoy et al.[6]. ClayFF offers a robust parameterization for many minerals and their interfaces to aqueous solutions. Parameters for formic acid and formate are taken from GAFF[20] and par-

tial point charges have been optimized using a RESP fit.[21] It has been shown that point charges obtained in this way are usually compatible with ClayFF and other force fields which use reduced partial charges instead of the formal oxidation states, e.g. by comparison to energy-distance curves obtained from DFT.[22] In the literature this approach was used successfully for describing the interaction between magnetite and adsorbates like water and small organic molecules,[22,23] and various other types of oxides, e.g. iron oxyhydroxides,[24] alumina,[25] silica[26,27] and titania.[28,29] For solids with ionic character such as magnetite, it is often sufficient to represent the potential energy only by simple, non-bonded interactions, *i.e.*, electrostatic and van der Waals interactions. Van der Waals interactions are usually modeled by a Lennard-Jones potential, while Coulomb's law between point charges is used for electrostatic interactions:

$$V_{\mathrm{non-bonded}} = \sum_{ij} 4\epsilon_{ij} \left[\left(\frac{\sigma_{ij}}{r_{ij}} \right)^{12} - \left(\frac{\sigma_{ij}}{r_{ij}} \right)^{6} \right] + \frac{q_i q_j}{r_{ij}} \tag{SI1}$$

q_i and q_j denote the atomic partial point charges between interacting pairs of atoms i and j. Distances between interacting atoms are denoted by r_{ij}. ϵ_{ij} indicates how strongly these atoms attract each other and $\sqrt[6]{2}\sigma_{ij}$ corresponds to the distance at which the potential between the atoms has its minimum. Pair coefficients ϵ_{ij} and σ_{ij} for interacting atom pairs are calculated by standard Lorentz-Berthelot mixing rules: $\epsilon_{ij} = \sqrt{\epsilon_i \epsilon_j}$, $\sigma_{ij} = \frac{1}{2}(\sigma_i + \sigma_j)$.

Surface hydroxyl groups as well as carboxylic acids usually require the involvement of bonded interactions. Bonded interactions are commonly defined by harmonic bonds and angles, dihedrals and impropers. Since improper angles can be described by the same functional form as dihedrals, the full energy expression of intramolecular interactions usually has the following functional form:

$$V_{\mathrm{bonded}} = \sum_{\mathrm{bond}} k_{\mathrm{bond}} (r - r_{\mathrm{eq}})^2 + \sum_{\mathrm{angle}} k_{\theta} (\theta - \theta_{\mathrm{eq}})^2 + \sum_{\mathrm{dihedrals}} k_{\phi} [1 + d \cos(n\phi)] \tag{SI2}$$

The first term represents a harmonic bond where k_{bond} is the bond coefficient and r_{eq} is

the equilibrium bond length. The second term is a harmonic angle where k_θ is the angle coefficient and θ_{eq} is the equilibrium bond angle. The last term is a dihedral or improper torsion. The latter is used to preserve the planarity of a molecule, such as that found in formic acid. k_ϕ is the dihedral/improper coefficient, d is the sign convention, n is the non-negative integer coefficient, and ϕ is the dihedral or out-of-plane angle spanned by the four atoms involved. The total potential energy of a system of N atoms with coordinates $\{\mathbf{r}^N\}$ is the sum of bonded and non-bonded interactions $V(\{\mathbf{r}^N\}) = V_{\text{bonded}} + V_{\text{non-bonded}}$.

Table SI1: Force field parameters of magnetite and formate.

Atom type	ϵ_i / kcal mol^{-1}	σ_i / Å	q / e
(Fe^{2+})[a]	$9.0298 \cdot 10^{-7}$	4.90620	1.050
(Fe^{3+})[c]	$9.0298 \cdot 10^{-7}$	4.90620	1.575
(O)[c]	0.1554	3.16554	-1.050
carboxylic oxygen (O_C)[b]	0.1463	3.04812	-0.804
carboxylic carbon (C_C)[b]	0.0988	3.31521	0.731
carboxylic hydrogen (H_C)[b]	0.0161	2.44730	-0.123
hydroxyl oxygen (O_H)	0.1554	3.16554	-1.000
hydroxyl hydrogen (H_O)[c]	0.0000	0.00000	0.425
**Bond stretch**	k_{bond} / kcal mol^{-1}Å^{-2}	r_{eq} / Å	
$O_H - H_O$[c]	554.1349	1.000	
$C_C - O_C$[b]	652.5700	1.218	
$C_C - H_C$[b]	361.8000	1.105	
**Angle bend**	k_θ / kcal mol^{-1}	θ_{eq} / deg	
$Fe - O_H - H_O$[c]	30.000	109.47	
$O_C - C_C - O_C$[b]	118.817	130.25	
$O_C - C_C - H_C$[b]	65.930	123.65	
**Improper angle**	k_ϕ / kcal mol^{-1}	sign	integer
$C_C - O_C - O_C - H_C$[b]	10.5	-1	2

[a] Taken from Gürsoy et al.[6]
[b] Taken from Konuk et al.[22]
[c] Taken from Cygan et al.[19]

Electron Localization Function

Density functional theory (DFT) calculations were performed on small model systems for investigating the bonds between the adsorbate and the substrate. The electron localisation function (ELF)[30,31] was employed for analysing the bonds. The ELF is a measure of electron

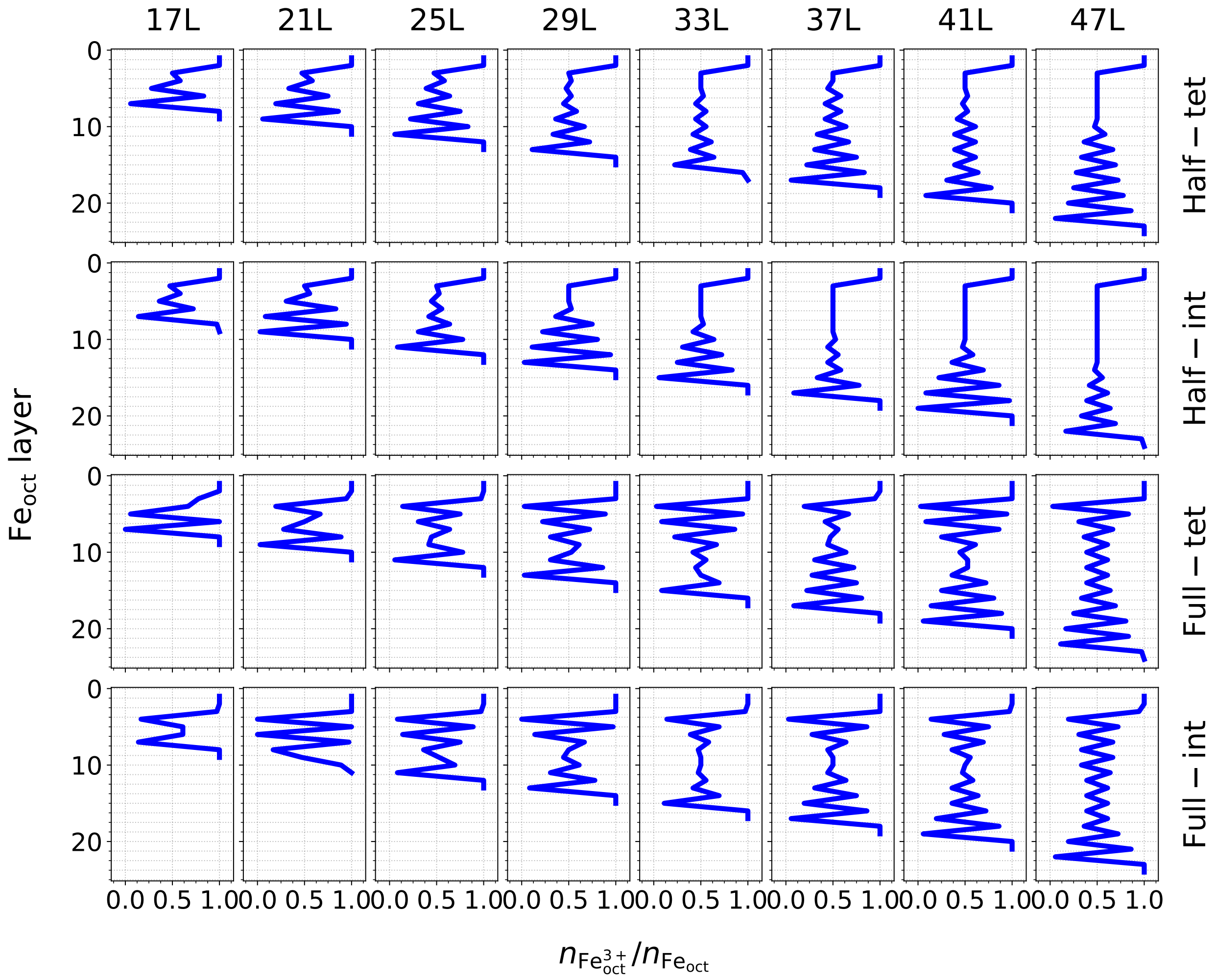

Figure SI1: **Minimized oxidation states of formate coated magnetite slabs.** One side of the slab is coated. The Fe^{3+}_{oct} ratio within each octahedral layer is denoted by $n^{3+}_{Fe_{oct}}/n_{Fe_{oct}}$. The surface layer where adsorbates are located corresponds to Fe_{oct} layer $= 1$. The surface thickness of each model, represented by (Fe_{oct}+Fe_{tet}), is given in the title, where "L" is abbreviation of "Layer". Binding sites and coverage ratios are given on the right side.

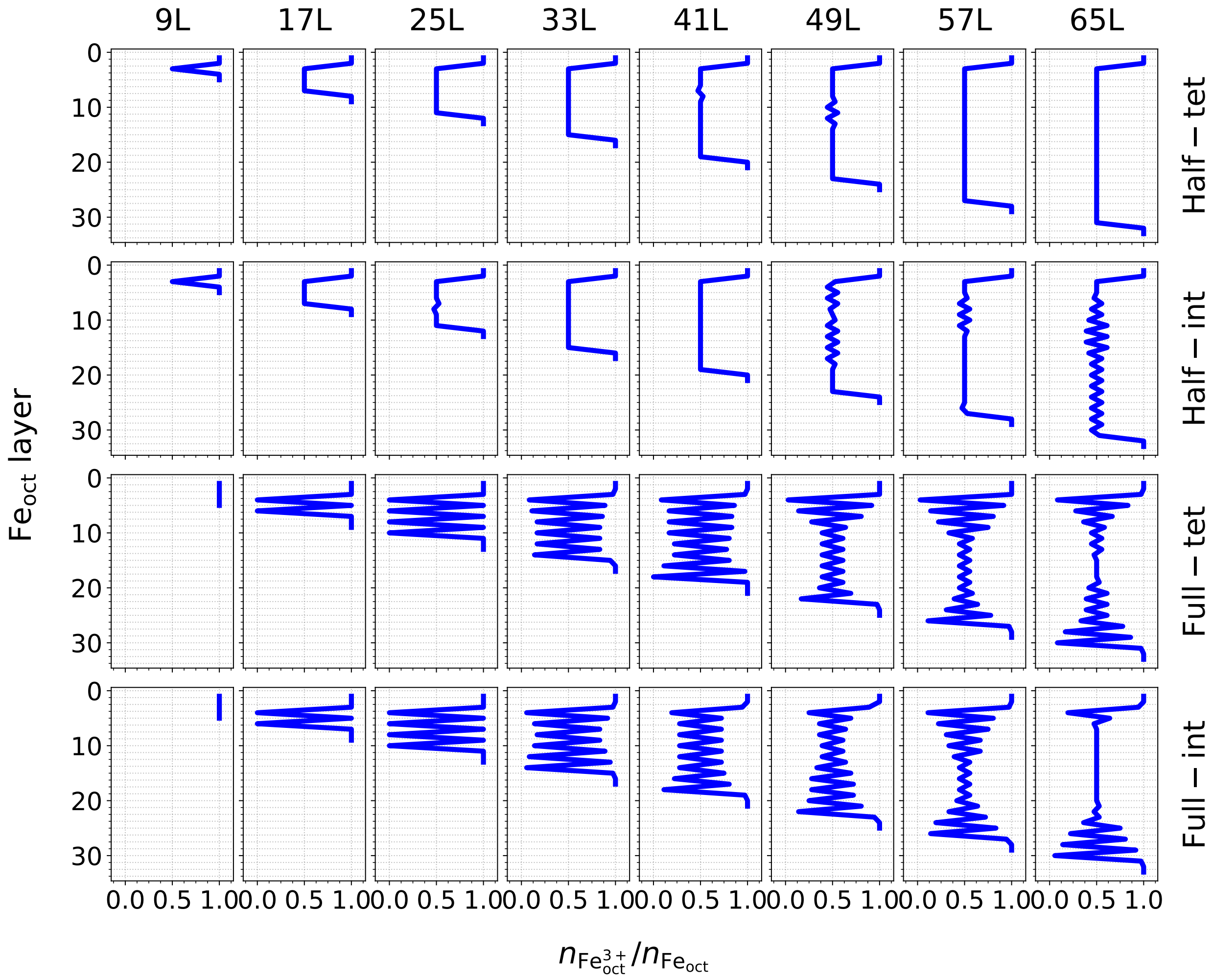

Figure SI2: **Minimized oxidation states of formate coated magnetite slabs with OH groups at both top and bottom interfaces are facing the opposite direction.** The Fe^{3+}_{oct} ratio within each octahedral layer is denoted by $n^{3+}_{Fe_{oct}}/n_{Fe_{oct}}$. The surface thickness of each model, represented by (Fe_{oct}+Fe_{tet}), is given in the title, where "L" is abbreviation of "Layer". Binding sites and coverage ratios are given on the right side.

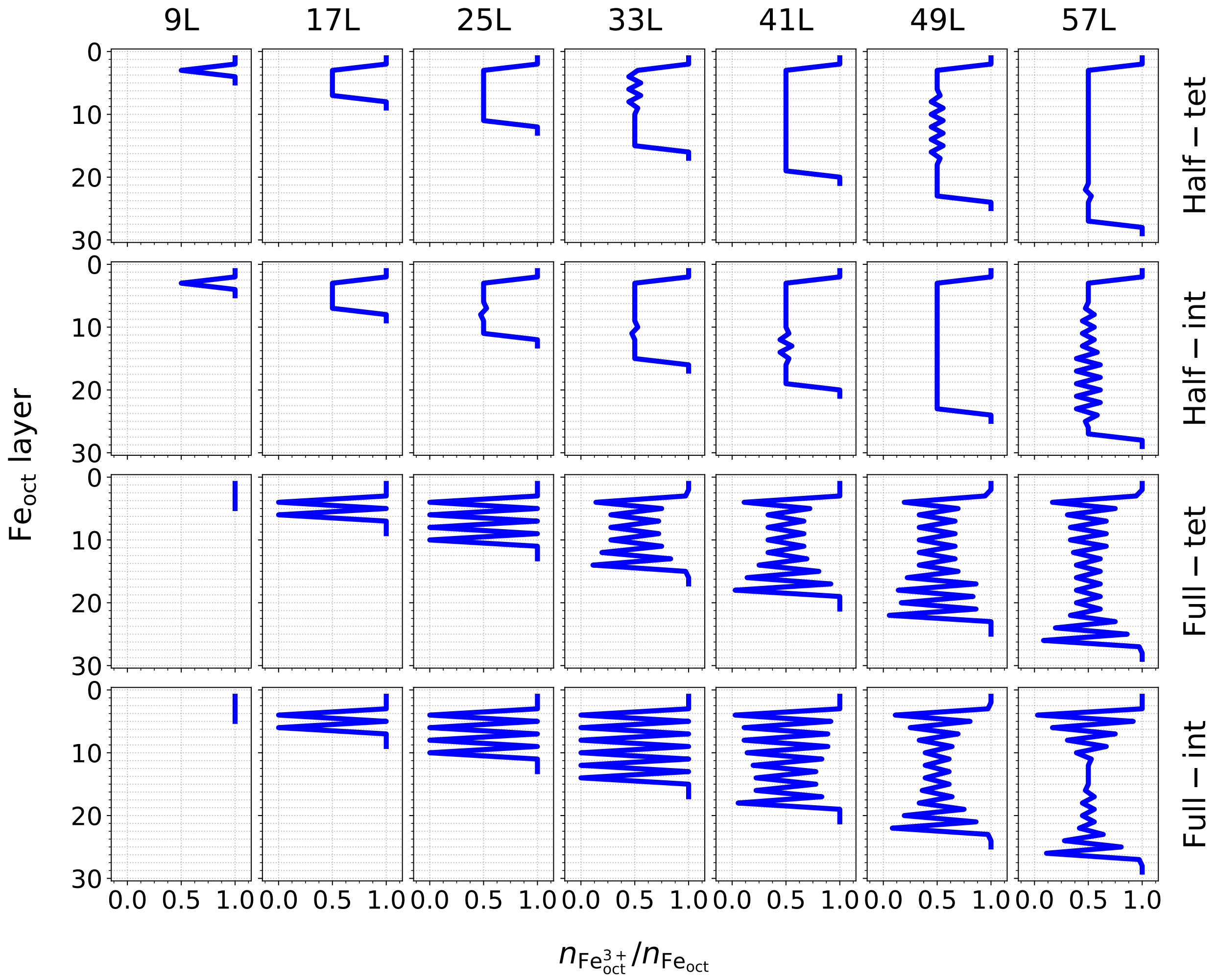

Figure SI3: **Minimized oxidation states of formate coated magnetite slabs on both sides with OH groups at both top and bottom interfaces are facing the same direction.** The Fe^{3+}_{oct} ratio within each octahedral layer is denoted by $n^{3+}_{Fe_{oct}}/n_{Fe_{oct}}$ The surface thickness of each model, represented by (Fe_{oct}+Fe_{tet}), is given in the title, where "L" is abbreviation of "Layer". Binding sites and coverage ratios are given on the right side.

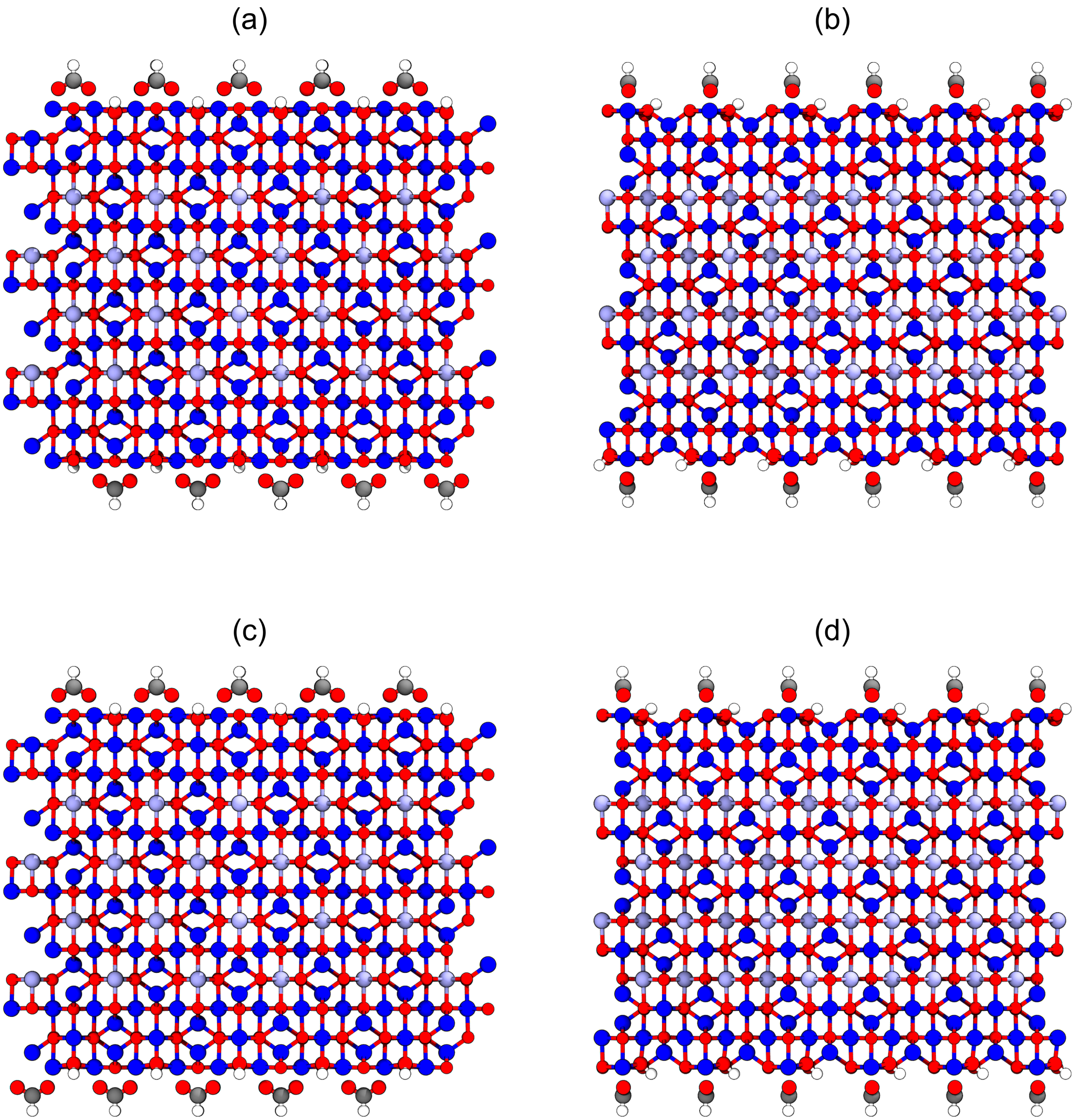

Figure SI4: **Oxidation state minimized 17L thick magnetite slab with full coverage.** (a) Front view with both OH groups at the interface (top and bottom) having the same direction and (b) side view. (c) Front view with OH groups having the opposite direction and (d) side view. Color code: Fe^{3+}_{oct} – dark blue, Fe^{2+}_{oct} – ice blue, O – red, H – white, C – gray .

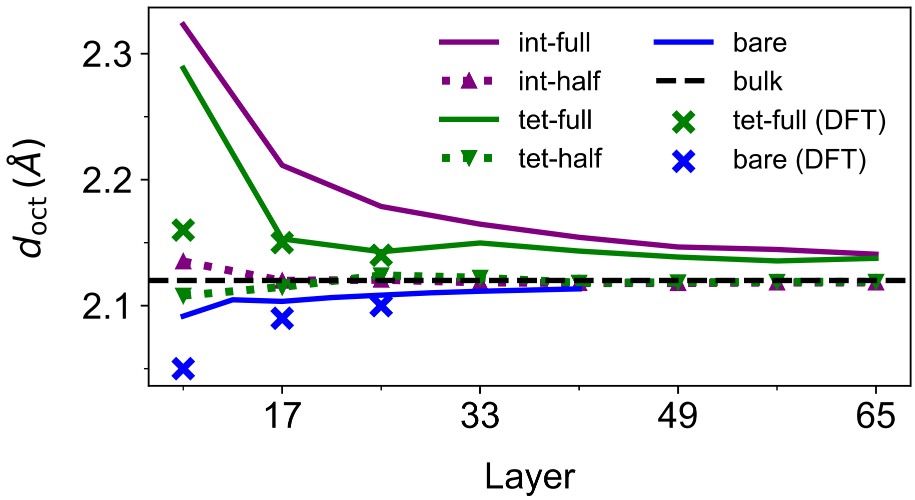

Figure SI5: **The average distance between octahedral layers (d_{oct}) as a function of surface thickness.** d_{oct} values of oxidation state minimized structures at half and full coverage ("tet" and "int" binding sites), bare surface, and bulk magnetite are shown, with DFT values given for comparison.

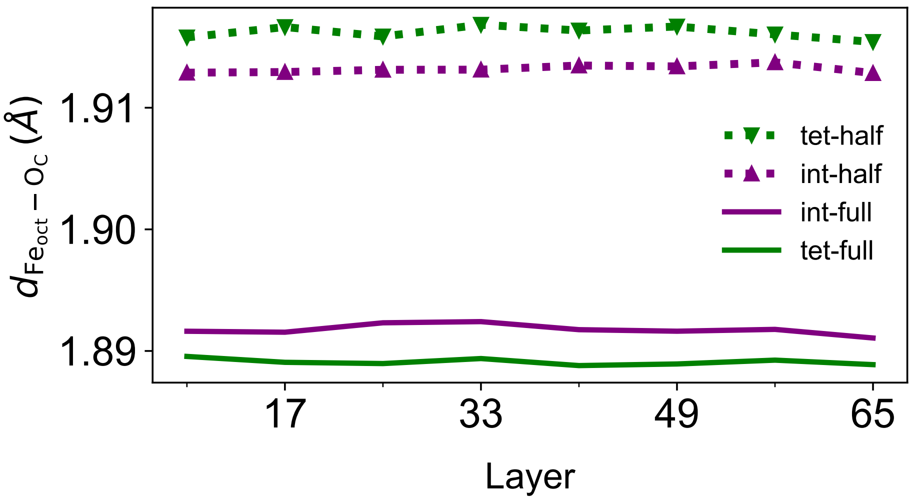

Figure SI6: **The average distance between surface Fe_{oct} ions and formate oxygens (O_C), as a function of surface thickness.** $d_{Fe_{oct}-O_C}$ values of oxidation state minimized structures at half and full coverage ("tet" and "int" binding sites) are shown.

localisation. While a value of 0.5 corresponds to the localisation of the uniform electron gas, a value of 1 means perfect localisation. This means a covalent bond is characterised by a high ELF value between the atoms involved in the bond. In Fig. SI7, the high ELF values between the C and O atoms in the formate indicate covalent bonding. At the formate-Fe_3O_4 interface, the electrons are however localised close to the acid O atoms without a common ELF isosurface including the next Fe atom, arguing against a covalent bond. According to a Bader charge analysis, using the implementation of Henkelman *et al*,[15–17] the slab Fe atoms are positively charged, while the formate has a negative charge. This suggests mainly an electrostatic interaction between the surface Fe and the formate.

Table SI2: Binding site preference (tet, int) of formic acid on (001) surface obtained from DFT.

Structure	$\Delta E_{\text{tet-int}}$ (eV/formate)
9L, half covered	0.001
9L, fully covered	-0.049
17L, half covered	0.026
17L, fully covered	-0.047
25L, half covered	0.028

References

(1) Plimpton, S. Fast parallel algorithms for short-range molecular dynamics. *Journal of computational physics* **1995**, *117*, 1–19.

(2) Hockney, R. W.; Eastwood, J. W. *Computer simulation using particles*; crc Press, 2021.

(3) Yeh, I.-C.; Berkowitz, M. L. Ewald summation for systems with slab geometry. *The Journal of chemical physics* **1999**, *111*, 3155–3162.

(4) Cornell, W. D.; Cieplak, P.; Bayly, C. I.; Gould, I. R.; Merz, K. M.; Ferguson, D. M.; Spellmeyer, D. C.; Fox, T.; Caldwell, J. W.; Kollman, P. A. A second generation force

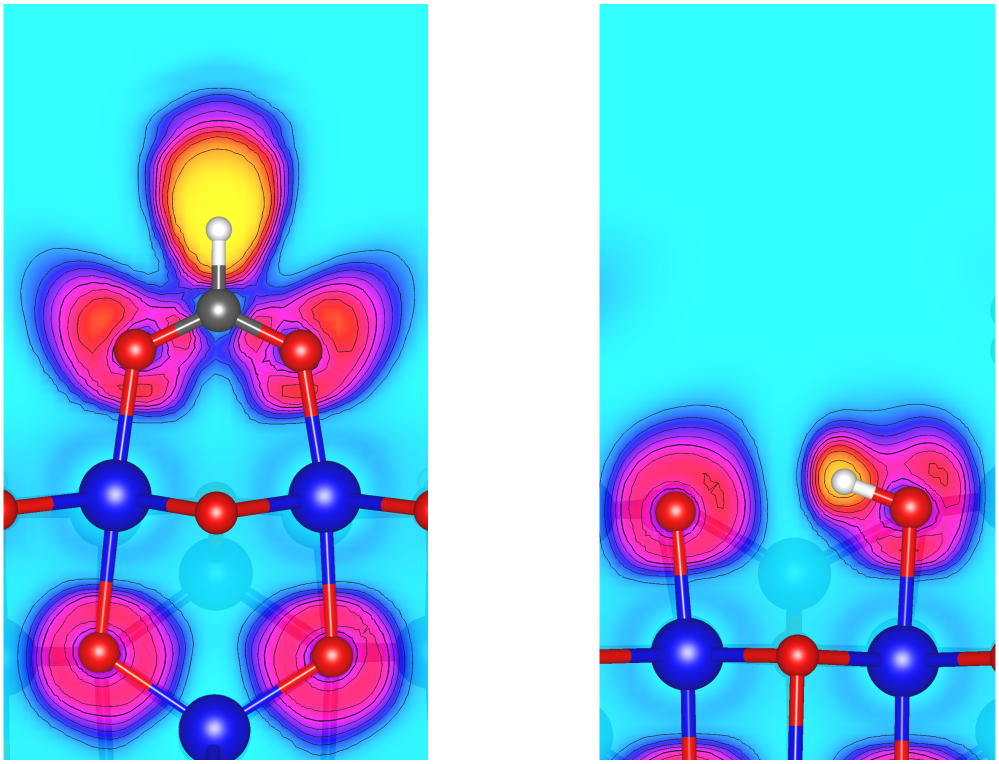

Figure SI7: **Illustration of the Electron Localization Function (ELF) for the equilibrium strcuture of formic acid on the Fe_3O_4(001) surface after dissociative adsorption.** Isosurfaces of the ELF are shown starting from the value of 0.0 to 0.9 in 0.1 steps and coloured from cyan to yellow. Magenta corresponds to a value of 0.5. In the left and right subfigures sections through the ELF parallel to the Fe-O-C-O-Fe bonds and an O-H bond, respectively, are shown. *Color code of elements:* Fe – blue, O – red, C – grey, H – white.

field for the simulation of proteins, nucleic acids, and organic molecules. *Journal of the American Chemical Society* **1995**, *117*, 5179–5197.

(5) Arndt, B.; Sellschopp, K.; Creutzburg, M.; Grånäs, E.; Krausert, K.; Vonk, V.; Müller, S.; Noei, H.; Feldbauer, G. B.; Stierle, A. Carboxylic acid induced near-surface restructuring of a magnetite surface. *Communications chemistry* **2019**, *2*, 1–8.

(6) Gürsoy, E.; Vonbun-Feldbauer, G. B.; Meißner, R. H. Oxidation-State Dynamics and Emerging Patterns in Magnetite. *The Journal of Physical Chemistry Letters* **2023**, *14*, 6800–6807.

(7) Kresse, G.; Furthmüller, J. Efficiency of ab-initio total energy calculations for metals

and semiconductors using a plane-wave basis set. *Computational Materials Science* **1996**, *6*, 15–50.

(8) Kresse, G.; Furthmüller, J. Efficient iterative schemes for ab initio total-energy calculations using a plane-wave basis set. *Physical Review B* **1996**, *54*, 11169–11186.

(9) Kresse, G.; Hafner, J. Ab initio molecular dynamics for liquid metals. *Physical Review B* **1993**, *47*, 558–561.

(10) Kresse, G.; Hafner, J. Ab initio molecular-dynamics simulation of the liquid-metalamorphous- semiconductor transition in germanium. *Physical Review B* **1994**, *49*, 14251–14269.

(11) Perdew, J. P.; Burke, K.; Ernzerhof, M. Generalized Gradient Approximation Made Simple. *Phys. Rev. Lett.* **1996**, *77*, 3865–3868.

(12) Dudarev, S. L.; Botton, G. A.; Savrasov, S. Y.; Humphreys, C. J.; Sutton, A. P. Electron-Energy-Loss Spectra and the Structural Stability of Nickel Oxide: An LSDA+U Study. *Phys. Rev. B* **1998**, *57*, 1505–1509.

(13) Blöchl, P. E. Projector Augmented-Wave Method. *Phys. Rev. B* **1994**, *50*, 17953–17979.

(14) Liu, H.; Di Valentin, C. Band Gap in Magnetite above Verwey Temperature Induced by Symmetry Breaking. *J. Phys. Chem. C* **2017**, *121*, 25736–25742.

(15) Henkelman, G.; Arnaldsson, A.; Jónsson, H. A fast and robust algorithm for Bader decomposition of charge density. *Comput. Mater. Sci.* **2006**, *36*, 354–360.

(16) Sanville, E.; Kenny, S. D.; Smith, R.; Henkelman, G. Improved grid-based algorithm for Bader charge allocation. *J. Comput. Chem.* **2007**, *28*, 899–908.

(17) Tang, W.; Sanville, E.; Henkelman, G. A grid-based Bader analysis algorithm without lattice bias. *J. Phys. Condens. Matter* **2009**, *21*, 084204.

(18) Kirkpatrick, S.; Gelatt, C. D.; Vecchi, M. P. Optimization by Simulated Annealing. *Science* **1983**, *220*, 671–680.

(19) Cygan, R. T.; Liang, J.-J.; Kalinichev, A. G. Molecular models of hydroxide, oxyhydroxide, and clay phases and the development of a general force field. *The Journal of Physical Chemistry B* **2004**, *108*, 1255–1266.

(20) Wang, J.; Wolf, R. M.; Caldwell, J. W.; Kollman, P. A.; Case, D. A. Development and testing of a general amber force field. *Journal of Computational Chemistry* **2004**, *25*, 1157–1174.

(21) Cornell, W. D.; Cieplak, P.; Bayly, C. I.; Kollman, P. A. Application of RESP charges to calculate conformational energies, hydrogen bond energies, and free energies of solvation. *Journal of the American Chemical Society* **1993**, *115*, 9620–9631.

(22) Konuk, M.; Sellschopp, K.; Vonbun-Feldbauer, G. B.; Meißner, R. H. Modeling charge redistribution at magnetite interfaces in empirical force fields. *The journal of physical chemistry C* **2021**, *125*, 4794–4805.

(23) Siani, P.; Bianchetti, E.; Di Valentin, C. Building up accurate atomistic models of biofunctionalized magnetite nanoparticles from first-principles calculations. *npj Computational Materials* **2025**, *11*.

(24) Larrucea, J.; Lid, S.; Colombi Ciacchi, L. Parametrization of a classical force field for iron oxyhydroxide/water interfaces based on Density Functional Theory calculations. *Computational Materials Science* **2014**, *92*, 343–352.

(25) Lid, S.; Köppen, S.; Colombi Ciacchi, L. Creation of models and parametrization of a classical force field for amorphous Al_2O_3/water interfaces based on Density Functional Theory. *Computational Materials Science* **2017**, *140*, 307–314.

(26) Butenuth, A.; Moras, G.; Schneider, J.; Koleini, M.; Köppen, S.; Meißner, R.; Wright, L. B.; Walsh, T. R.; Ciacchi, L. C. *Ab initio* derived force-field parameters for molecular dynamics simulations of deprotonated amorphous-SiO_2/water interfaces. *physica status solidi (b)* **2011**, *249*, 292–305.

(27) Meißner, R. H.; Wei, G.; Ciacchi, L. C. Estimation of the free energy of adsorption of a polypeptide on amorphous SiO_2 from molecular dynamics simulations and force spectroscopy experiments. *Soft Matter* **2015**, *11*, 6254–6265.

(28) Schneider, J.; Ciacchi, L. C. A Classical Potential to Model the Adsorption of Biological Molecules on Oxidized Titanium Surfaces. *Journal of Chemical Theory and Computation* **2010**, *7*, 473–484.

(29) Schneider, J.; Colombi Ciacchi, L. Specific Material Recognition by Small Peptides Mediated by the Interfacial Solvent Structure. *Journal of the American Chemical Society* **2012**, *134*, 2407–2413.

(30) Becke, A.; Edgecombe, K. A Simple Measure of Electron Localization in Atomic and Molecular Systems. *J. Chem. Phys.* **1990**, *92*, 5397–5403, cited By (since 1996)1672.

(31) Silvi, B.; Savin, A. Classification of Chemical Bonds Based on Topological Analysis of Electron Localization Functions. *Nature* **1994**, *371*, 683–686.
